# Financial toxicity in lung cancer

**DOI:** 10.3389/fonc.2022.1004102

**Published:** 2022-10-21

**Authors:** Mary Boulanger, Carley Mitchell, Jeffrey Zhong, Melinda Hsu

**Affiliations:** ^1^ Department of Medicine, Johns Hopkins University, Baltimore, MD, United States; ^2^ Department of Oncology, University Hospitals Seidman Cancer Center, Cleveland, OH, United States; ^3^ Department of Medicine, Case Western Reserve University School of Medicine, Cleveland, OH, United States

**Keywords:** financial toxicity, lung cancer, ASCO value framework, quality of life, survivorship care

## Abstract

In the United States, lung cancer is the third most common cancer and the overall leading cause of cancer death. Due to advances in immunotherapy and targeted therapy, 5-year survival is increasing. The growing population of patients with lung cancer and cancer survivors highlights the importance of comprehensive cancer care, including recognizing and addressing financial toxicity. Financial toxicity is a term used to contextualize the negative effects of the costs of cancer treatment in terms of patient quality of life. The American Society of Clinical Oncology (ASCO) Value Framework places emphasis on high-value care as it evaluates cancer treatments “based on clinical benefit, side effects, and improvements in patient symptoms or quality of life in the context of cost”. Prior studies have shown that risk factors for financial toxicity in patients with lung cancer include lower household income or savings, inability to afford basic necessities, higher than anticipated out of pocket expenses, and taking sick leave. Among lung cancer survivors, patients experience increased unemployment and lower wages compared to the general population underscoring the lasting effects of financial toxicity. Financial toxicity is associated with increased psychosocial distress and decreased quality of life, and bankruptcy is an independent predictor of mortality in patients with cancer. Despite the negative implications of financial toxicity on patients, standardized screening practices and evidence-based interventions are lacking. The “COmphrensive Score for financial Toxicity (COST)” tool has been validated for assessing financial toxicity with correlation with health-related quality of life. Further research is needed to understand the utility of incorporating routine screening for financial toxicity into clinical practice and the efficacy of interventions. Understanding the relationship between financial toxicity and quality of life and survival is critical to providing high-value cancer care and survivorship care.

## Introduction

Despite a consistent decline in incidence and mortality over the past two decades, lung cancer continues to remain the leading cause of cancer-related deaths among both men and women in the United States (1). With only a 22% 5-year survival rate (1), great effort has been focused on the development of new treatment approaches and detection strategies. While many of these advancements offer hope for improved patient outcomes, unforeseen physical and socioeconomic side effects often emerge.

Financial toxicity refers to issues caused by the cost of medical care (2) and is characterized by the monetary burden, poor outcomes, and psychological distress impacting patients (3). Financial difficulties not only stem from high-cost medical treatment and diagnostics, but also non-medical costs such as transportation, parking, lodging and caregiving, as well as indirect costs from lost income and wages (4). While the concept of financial toxicity is not new, its impact has become increasingly prominent as the cost of living and cancer care continues to rise. Based on estimates from the National Cancer Institute, overall cancer-related medical costs will increase greater than 34% from the year 2015 to 2030 (4). While private and government-funded insurance programs will absorb much of this cost, higher deductibles, co-payments and out-of-pocket (OOP) expenses will undoubtedly fall to the patient. OOP expenses in 2018 accounted for 5%, or 5.6 billion dollars, of total cancer-related treatment expenses (4). The annual per-patient cost of medical services for patients with lung cancer ranges from 12.2 to 118 thousand dollars annually, with the greatest financial burden occurring at the time of initial diagnosis and the last year of life (5).

Financial hardship does not impact all patients equally and may wax and wane throughout a lifetime. Those more likely to suffer include patients of younger age, minority status, minimal educational experience, and those with decreased household savings and inability to afford basic necessities (4, 6). Because of this financial instability, negative outcomes can be seen on physical, mental, emotional, and economic levels (4). Physical health may be sacrificed in an effort to save money by delaying medical appointments or foregoing medications (7). In addition, significant stress and worry regarding individual health and finances can result in poor mental health outcomes, and accrual of monetary debt may lead to food and housing insecurity along with bankruptcy (8). These effects take a significant toll on individual quality of life (QOL) and wellbeing with the potential to worsen symptom burden and hasten patient mortality (6, 8). Focused attention at the patient, clinician, professional society and governmental level is needed to address and counteract this complex area of medical care. The focus of this review will be to highlight prior research, contributing factors, and potential interventions to address financial toxicity in patients with lung cancer.

## Discussion

### Financial toxicity in patients with cancer

As the United States (US) population ages alongside increasing cancer survival rates, a growing number of individuals with cancer will be impacted by financial toxicity. A study conducted by Mariotto et al. offered projections of increasing cancer prevalence and estimated care costs from the years 2010 to 2020. They estimated a rise in cancer prevalence from 13.7 million to 18 million, with a projected 27% increase in national expenditure solely based on the growing and aging US population, while keeping current cancer incidence, survival and cost constant (9). It is not surprising that cost varies by disease and throughout an individual disease course, which was also considered in the above estimation. For instance, colorectal cancer produces the highest cost during the initial phase of the disease, with lung cancer accounting for the highest costs during the final year of life, and prostate/breast cancers creating the highest expenditure during the middle, continuing phase of care (9). With an ever-growing increase in cancer prevalence, understanding the impact and determining ways to combat the effects of financial toxicity is imperative.

Various outcomes of previous financial toxicity research among the general population with cancer were nicely summarized by Altice et al. in a systematic review of 45 studies between 1990-2015. The estimated monthly OOP costs for patients ranged from $316 to $741, and most studies emphasized that those with a cancer diagnosis faced significantly higher OOP costs compared to those without (10). Not only were direct costs higher, but indirect costs from lost days at work or decreased productivity ranged from $380 to $8,236, annually (10). Additionally, 2-3% of individuals diagnosed with cancer filed bankruptcy claims within the first two years of diagnosis, and a vast majority of patients utilized income or savings to pay for medical expenses while 7-10% increased credit card debt or borrowed money from family or friends (10). Multiple studies found that effects of financial instability led to difficulty affording necessities such as clothes, food, and home utilities (10). Patients with cancer were also more apt to avoid spending on other areas or healthcare including prescription refills and experienced increased rates of stress with a greater risk of depression compared to the general population (10). Lack of transportation leads to delays in care, especially in patients who are single, have lower income, are underinsured, or have self-reported physical limitations (11). Furthermore, the cumulative costs of parking alone for cancer related appointments is burdensome, with median parking cost of $2 per hour or $5 per day at National Cancer Institute-Designated Cancer Treatment Centers (12). Only 54% of NCI-Designated Treatment Centers have free parking available for chemotherapy appointments (12).

A survey study of 1,202 adult cancer survivors in the US explored material and psychological hardship associated with cancer. One fifth of survivors experienced material financial hardship, and almost a quarter of survivors experienced psychological hardship. Younger patients, defined as between 18 to 64 years, experienced a statistically significant increase in both material and psychological hardship related to financial toxicity compared to patients ≥ 65 years (13). Among younger patients, material hardship was associated with female gender and undergoing more recent treatment (13). Interestingly, psychological hardship was more common in younger patients who were uninsured compared to private insurance but did not vary by type of insurance in the older patients (13).

### Measures of financial toxicity

While greater attention has been paid to financial toxicity in recent years, identifying and validating standardized means of measurement remains an area of ongoing research. A systematic review conducted by Witte et al. evaluated 43 studies plus six systematic reviews from 2006 to 2018 highlighting various measurement tools. Most studies hailed from the United States with a majority encompassing all cancer types, with six studies focusing solely on lung cancer (14). Primary means of measurement were in the form of patient-reported questionnaires (14). Three broad domains of financial toxicity and their subtypes were described: material conditions, further broken down into active spending and the passive utilization of personal financial resources; psychological response represented by the patient’s affect; and coping behaviors, further divided into support seeking behaviors, care plan adjustments, and lifestyle modifications (14).

Some individual studies within the Witte et al. review focused their questionnaire on overall health-related quality of life (HRQoL) with only a subset of questions targeting the patient’s financial situation. These included the European Organization for Research and Treatment of Cancer Core Quality of Life Survey (EORTC QLQ-C30), Cancer Care Outcomes Research and Surveillance Consortium Patient Survey (CanCORS), Social Difficulties Inventory (SDI) and the Patient Satisfaction Questionnaire (PSQ-18) (14). EORTC QLQ-C30 is one of the most frequently used tools to assess cancer-related QOL (15) *via* 30 questions focusing on functional status, physical symptoms and perceived QOL, with only one question addressing financial difficulties (14). Similarly, the CanCORS patient survey also incorporates one question regarding financial burden (14). Specifically, it asks, “If you lost all of your current sources of income (for example, paycheck, Social Security, pension, public assistance) and had to live off of your savings, how long could you continue to live at your current address and standard of living?” (16). Unlike EORTC QLQ-C30, this survey has only been studied among patients with lung and colorectal cancers and the above question has shown an independent association between low levels of financial reserve and poorer QOL along with higher symptom burden (16). The SDI is a 21-question survey developed to measure a variety of social issues impacting patients diagnosed with cancer including personal care, work, family matters, communication, etc. with two questions focusing on financial issues (17). While the EORTC QLQ-C30, CanCORS and SDI are all cancer-specific questionnaires, the PSQ-18 is often used to assess a broad patient population including but not limited to those with cancer (14). The PSQ-18 is an 18-item questionnaire addressing overall patient satisfaction among various facets of care with one domain being “financial aspects” (18). One study, not included in the Witte et al. review, evaluated financial toxicity among patients with cancer utilizing the following PSQ-18 item; “You have to pay for more medical care than you can afford” with corresponding Likert scale responses of strongly agree, agree, uncertain, disagree, and strongly disagree (18). Those who chose strongly agree or agree were deemed to exhibit financial toxicity (18). Results from this study showed similar patient demographics and overall prevalence of financial toxicity among the study population compared to previously reported data, suggesting that this question may be an appropriate screening tool to quickly identify at-risk patients (18). Developing efficient, valid and reliable means of screening is an important aspect of financial toxicity to allow for early intervention at, or before, the initiation of treatment. Future studies comparing individual questions, such as those utilized in the above questionnaires, are needed to help better address this area.

Other studies within the Witte et al. review utilized multi-item questionnaires which were designed to specifically assess subjective financial distress including The Comprehensive Score for Financial Toxicity (COST), Breast Cancer Finances Survey Inventory (BCFS), Socioeconomic Wellbeing Scale (SWBS) and InCharge Financial Distress/Financial Well-Being Scale (IFDFW) (14). The IFDFW is the only generic tool among those listed and focuses on psychosocial affect, financial resources, and coping strategies (14). This scale is identified as a valid and reliable tool to measure financial distress among individuals in a vast array of settings including healthcare (19). However, due to the unique financial burdens of those with cancer compared to those with other chronic medical conditions and moreover, the general population, a more focused means of measurement is preferable. The BCFS tool has questions encompassing all three financial toxicity domains but only four of the six subtypes outlined by Witte et al., including financial spending, utilization of financial resources, patient affect and lifestyle modifications (14). BCFS was developed specifically for utilization among patients with breast cancer, making its generalizability to other cancer types – such as lung cancer – limited (19). The SWBS was originally developed as a subscale for other questionnaires focused on overall HRQoL, however it can be used independently as well (14). The questions are skewed toward the material domain of financial toxicity including financial spending and utilization of financial resources with questions regarding care plan adjustments and psychosocial affect also included (14); utilization of this measurement tool is limited.

Lastly, the COST tool, developed in 2014, is one of the most widely used instruments to assess financial toxicity in patients with cancer (20). It is an 11-item patient-reported outcome measure with a large focus on the psychosocial domain of financial toxicity followed by financial resource utilization and financial spending (14). Due to significant need for a tool measuring financial toxicity at the time, COST was deployed in both research and clinical domains upon its development, even prior to establishing validity and reliability (21). COST measurements were significantly associated with employment status, race, income, psychological distress along with the number of inpatient admissions (22). This study was the first to demonstrate a positive association between financial toxicity and the frequency of inpatient admissions (22). Additionally, this study indicated a statistically significant correlation to HRQoL making this a clinically relevant tool as well (22). Not only has COST been validated in the United States but it has been validated in other countries with varying healthcare financing models (23–25). Scores for COST range from 0-44 with lower scores correlating to greater financial toxicity. While some studies have set COST thresholds based on percentiles obtained from their unique study population (21), De Souza et al. defined a grading system for financial toxicity utilizing the COST tool which has been studied among patients with various types of cancer demonstrating consistent validity (26). Grade 0 indicates absence of financial toxicity with a score of greater than or equal to 26 points. Grade 1 indicates mild financial toxicity with a score between 14-25. Grade 2 indicates moderate financial toxicity with a score 1-13, and grade 3 indicates severe financial toxicity with a score of zero (26). While the COST tool appears to be the most widely used and studied cancer-specific instrument to measure financial toxicity, it is not specific to lung cancer. In fact, only nine of the 100 patients involved in the initial assessment and analysis of this questionnaire had lung cancer diagnoses (27). It is, however, the most frequently used tool among studies specifically assessing financial toxicity in patients with lung cancer.

### Implications of financial toxicity in lung cancer care

Prior studies have described the risk factors for and consequences of financial toxicity in lung cancer care. Friedes and Hazell et al. conducted a prospective longitudinal study of 215 patients with stage II-IV lung cancer between July 2018 to May 2020 assessing COST at diagnosis and six-month follow up (28). At diagnosis, household income less than $40,000, having less than one month of savings, and inability to afford basic necessities were associated with financial toxicity (28). At six-month follow up, having less than one month of savings and inability to afford basic necessities were still associated with financial toxicity, as well as being on sick leave and paying more than anticipated for OOP costs. Interestingly, most patients at diagnosis over-estimated their OOP costs, with median reported costs $2496 compared to median estimates of $3000. At six-month follow up, 27.7% of patients reported making sacrifices to pay for medical care, including using personal savings or selling assets, borrowing money, or changing housing. Furthermore, 17.9% were unable to afford basic necessities, which was defined as “ability to pay for gas, electricity, bills, food, prescription medication, or other monthly structure payments”. However, only 4.5% reported withholding medical care due to cost. Only 9.8% of patients saw a financial counselor at diagnosis and 14.6% retrospectively reported that they wished they had. Overall, there was a small, statistically significant improvement in financial toxicity from diagnosis to the six-month mark, though 27.4% of patients did not have six-month follow up data due to research limitations during the COVID-19 pandemic. Friedes and Hazell et al. importantly demonstrated the evolution of financial toxicity in lung cancer treatment (28).

Financial toxicity is associated with decreased HRQoL in patients with stage III-IV lung cancer (29). Furthermore, financial distress requiring bankruptcy is a risk factor for early mortality (hazard ratio 1.79; 95% CI, 1.64 to 1.96) across a broad range of malignancies (8). While no patients in the study by Friedes and Hazell et al. declared bankruptcy, a study by Chino et al. conducted in 245 patients with solid tumors including 39 patients with lung cancer, found that 49% reported willingness to declare bankruptcy to afford medical care at baseline assessment and 42% at three-month follow up (30). Patients with lung cancer at 5-years from diagnosis had the highest cumulative incidence of bankruptcy and lowest overall survival compared to survivors of other malignancies (8). Among lung cancer survivors, patients experience increased unemployment and lower wages compared to the general population underscoring the lasting effects of financial toxicity (31).

Weaver et al. evaluated 6,602 adult cancer survivors (including breast, cervical, melanoma, prostate, or multiple cancers) and 104,364 individuals with no cancer history and demonstrated that among cancer survivors, 7.8% forego medical care and 9.9% forego prescription medications compared to 5.2% and 7.2% in the general population, respectively (32). Additionally, cancer survivors under age 65 were more likely to forego care. While this study was evaluating non-lung malignancies, it highlights the need to further characterize financial toxicity in lung cancer by age. The study by Friedes and Hazell et al. evaluating patients with lung cancer included only 7% of patients under 50 years old, highlighting a gap in current research in younger patients with lung cancer. Younger patients with cancer experience bankruptcy at higher rates among all cancer types, and patients with lung cancer were 3.8 times more likely to go bankrupt than controls in a study by Ramsey et al. While most patients diagnosed with lung cancer are age 65 or older (33), as lung cancer screening increases, we anticipate a decrease in the average age at diagnosis.

Meeker et al. evaluated overall distress and financial distress in 119 patients with solid malignancies, stratifying by age groups (defined as young <50, middle-age 50–64, and elderly ≥ 65 years of age) (34). The types of solid malignancies included were not specified. Overall distress was evaluated using the National Comprehensive Cancer Network (NCCN) distress thermometer and financial distress was measured by the IFDFW (34). In multivariable analysis, overall distress was most strongly associated with financial distress in middle aged patients (34).

While many of the advancements in lung cancer treatment based on cancer genomics offer incredible hope for improved survival, they can come at a high financial cost. Genomic testing alone can cost $300-$10,000 (35). Although testing with next generation sequencing is standard of care, the cost to patients is not readily available. Improved transparency about coverage of testing and OOP costs is needed for patients and clinicians. Furthermore, there is limited research regarding the cost of targeted therapies in patients with lung cancer. Skinner et al. evaluated 364 patients with advanced non-small cell lung cancer on tyrosine kinase inhibitors (TKI) (36). The mean monthly cost of systemic cancer therapy was $8,530 (95% CI $7,141–$9,919) for those who received TKI, accounting for 42.4% of their total mean monthly healthcare costs (36). Kaisaeng et al. evaluated patients with Medicare part D on oral cancer treatment, including 96 patients on erlotinib. Median OOP costs per day for erlotinib were $28.35, or $850.50 per month (37). For each $10 increase in OOP costs per month, the odds of discontinuation or delay increased 13.8% for those on erlotinib (37). Paying more than anticipated for OOP costs is associated with financial toxicity, and the lack of available information for patients regarding costs serves as a barrier to mitigating this risk.

Financial toxicity has major implications in terms of clinical trial enrollment. Although clinical trials are sometimes the best available treatment options for patients, only 5% of patients with cancer enroll in a clinical trial (38). Clinical trials often involve frequent travel, relocation, interruption of unemployment, and insufficient support to match expenses (38). Patients with lower income are less likely to participate in clinical trials (38). In order to provide equitable clinical trial access, appropriate financial incentives are needed to minimize the increased costs that may come with clinical trial participation. ASCO has called for improving the policy environment regarding coverage for clinical trials, targeted financial support, and greater transparency regarding the costs of clinical trial participation (39).

Numerous barriers to alleviating financial toxicity exist on a clinical, institutional, and systematic level. Lack of clinician expertise regarding costs of care and lack of time are important clinical limitations (40). Involvement of multidisciplinary teams, including financial counselors, social workers, case managers, nurse navigators, and pharmacists, is essential for comprehensive cancer care. The American Society of Clinical Oncology (ASCO) has called attention to high-value care through the Value Framework which evaluates cancer treatments “based on clinical benefit, side effects, and improvements in patient symptoms or quality of life in the context of cost” (41). Policy-level changes incentivizing high-value care are likewise needed to address financial toxicity in cancer care.

### Future directions

Despite the negative implications of financial toxicity on patients, standardized screening practices and evidence-based interventions are lacking. Further research is needed to understand the utility of incorporating routine screening for financial toxicity into clinical practice. The COST tool has been validated for assessing financial toxicity and also correlates with HRQoL (22). Utilizing the COST tool for screening in conjunction with targeted interventions by multidisciplinary teams to mitigate financial toxicity should be evaluated in both patients receiving active cancer treatment and cancer survivors. Additionally, continued research is needed to further understand financial toxicity differences among varying types of malignancies and treatment regimens with the potential for more targeted assessment tools. Furthermore, establishment of a cancer-specific instrument that equally accounts for all three domains and corresponding subtypes of financial toxicity, proposed by Witte et al., may be of great value.

Understanding and addressing the relationship between financial toxicity, QOL, and survival is critical to providing high-value cancer care and survivorship care. A longitudinal study of financial toxicity in patients with lung cancer is imperative to understanding the pervasive impact of cancer on patients. This is particularly relevant to understanding the experience of middle-aged patients, who are more likely to forego medical care and declare bankruptcy (32, 42). Additionally, research engaging the patient’s caregiver and family is needed to inform care discussions and planning. Active involvement of patient advocacy and research groups by healthcare systems, pharmaceutical companies, clinical trials, and legislators is critical to understanding how we can develop more patient-centric and less financially burdensome care.

## Conclusions

It is clear that individuals diagnosed with cancer are more vulnerable to the long-term effects of financial toxicity compared to the general population, and those with lung cancer appear to be at a particularly high risk (30, 32). Undoubtedly, this aspect of care is frequently overlooked due to other concerns such as treatment plans, imaging results, and complex symptom management. However, when patients are forced to make decisions to forego prescriptions, skip follow-up visits, or declare bankruptcy, the ability of the clinician to provide effective care is starkly limited and mortality rates rise. This not only affects patients receiving active treatment but likely impacts those in the survivorship phase as well, which may be a result of lost savings, increased unemployment, or lower wages leaving new challenges and worry in the place of cancer. Key areas of future focus include continued research and implementation of screening tools to identify those at risk, and effective utilization of multidisciplinary teams and care models to assess and develop individualized cost-conscious treatment methods. While this will help address clinician and institutional level approaches to combat this issue, a concerted effort must also be taken on a broader level to include insurance companies, pharmaceutical companies and medical governing bodies. With committed involvement of all stakeholders, the effects of financial toxicity can be limited while patient health and livelihood are enhanced.

## Author contributions

MB, CM, JZ, MH wrote and edited the manuscript. All authors contributed to the article and approved the submitted version.

## Conflict of interest

Author MH declares the following: Ad board/Consulting fees: Regeneron.

The remaining authors declare that the research was conducted in the absence of any commercial or financial relationships that could be construed as a potential conflict of interest.

## Publisher’s note

All claims expressed in this article are solely those of the authors and do not necessarily represent those of their affiliated organizations, or those of the publisher, the editors and the reviewers. Any product that may be evaluated in this article, or claim that may be made by its manufacturer, is not guaranteed or endorsed by the publisher.
